# Granulomatosis with polyangiitis presenting headache: A case report and review of literature

**DOI:** 10.1097/MD.0000000000036972

**Published:** 2024-01-12

**Authors:** Zhao Li, Qiushuang Zhang, Xuebin Wang, Fang Shi

**Affiliations:** a Department of Rheumatology, Binzhou Medical University Hospital, Binzhou, Shandong, China.

**Keywords:** case report, granulomatosis with polyangiitis, headache

## Abstract

**Rationale:**

Central nervous system involvement is a rare manifestation of active-phaselocalized Granulomatosis with polyangiitis (GPA). In hypertrophic dura meningitis, GPA with headache is typical. In this case, cerebral magnetic resonance (MR) enhancement revealed no meningeal thickening, to our knowledge, this manifestation had not been found previously.

**Patient concerns:**

The patient presented to the Rheumatology and Immunology Clinic with severe headache and hearing loss, and central nervous system granulomatosis with polyangiitis was confirmed after a series of examinations. The patient had no significant effect after treatment with cyclophosphamide (CTX), but after the use of rituximab, the headache and hearing loss were significantly improved, and laboratory indicators returned to normal levels.

**Diagnosis:**

We comprehensively screened for craniocerebral infection and malignant tumors, diagnosed central nervous system granulomatosis with polyangiitis.

**Interventions:**

We gave sequential treatment of rituximab.

**Outcomes:**

All indicators are mostly back to normal when the patient was monitored at the outpatient clinic.

**Lessons:**

GPA and severe headache are more prevalent in hypertrophic dura meningitis, but the patient early headache could not be explained by hypertrophic dura meningitis or localized granulomatous lesions that invaded the central nervous system. Patients with severe headaches likely have vascular inflammation and local bone destruction at the base of the skull.

## 1. Introduction

Granulomatosis with polyangiitis (GPA, formerly Wegener granuloma) is an immune-mediated condition characterized by necrotizing granulomas and necrotizing vasculitis affecting small and medium blood vessels and multiple organs and systems, including the upper and lower respiratory tract, kidneys, muscles, joints, and peripheral nerves.^[1]^ Neurological manifestations are present in 20% to 50% of GPA cases, with central nervous system involvement in 10% of patients.^[2,3]^ In hypertrophic dura meningitis, GPA with headache is typical.^[4]^ In this case, cerebral magnetic resonance (MR) enhancement revealed no meningeal thickening, to our knowledge, this manifestation had not been found previously. Therefore, a case of GPA with headache is reported in this study, alongside a review of relevant literature.

## 2. Case report

### 2.1. Patient information

**Figure 1.**
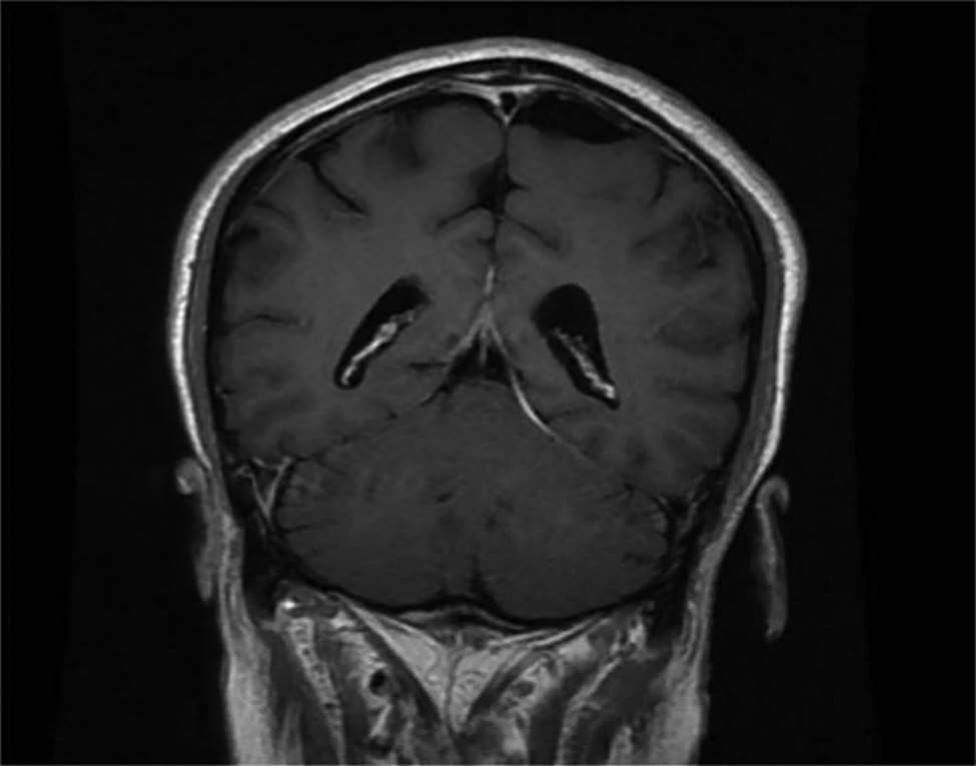
The MRI-enhanced T1 sequence revealed no dural thickening and abnormal enhancement. MRI = magnetic resonance imaging.

**Figure 2.**
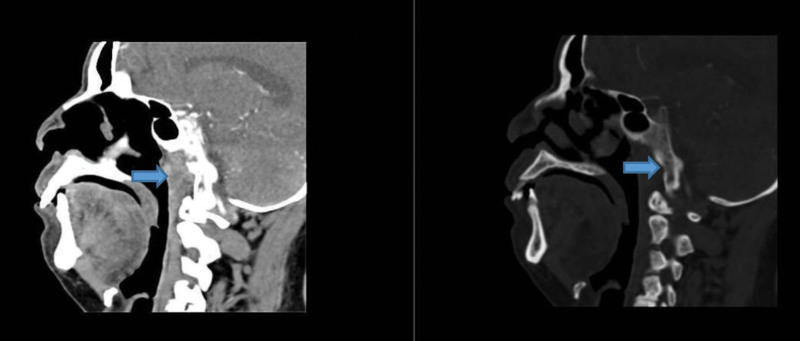
The presence of soft tissue surrounding the nasopharynx on a contrast-enhanced CT of the nasopharynx. CT = computed tomography.

A 63-year-old male was admitted to the hospital on October 5, 2022 with “lung occupancy for 1 year, headache for 1 month, hearing loss for 2 weeks.” The patient underwent particle implantation for “lung mass” and the interstitium of lung tissue was infiltrated and aggregated with more lymphocytes, plasma cells, and eosinophils as a result of postoperative pathology. One month before admission, the patient developed persistent left temporal pain with a visual analog scale (VAS) score of 10 and a feeling of tightness at the back of the neck. No fever, blurred vision, facial numbness, limb sensation, or movement disorders existed. She visited the local hospital for a battery of tests. Cranial magnetic resonance imaging (MRI) + magnetic resonance angiography + magnetic resonance venogram scans revealed no apparent abnormalities. The color ultrasound examination of the bilateral carotid arteries, bilateral vertebral arteries, and the heart revealed no apparent abnormalities. Laboratory tests reveal elevated erythrocyte sedimentation rate (ESR), C-reactive protein (CRP), white blood cells, and platelet counts. No abnormalities were observed in cerebrospinal fluid routine, biochemistry, or culture. The patient symptoms were relieved by oral prednisone acetate (60 mg/qd), but his headache worsened after the dosage was reduced to 30 mg/qd. Two weeks before admission, the patient experienced severe pain in the right temporal region and hearing loss in both ears, with a VAS score of 10 and no nasal congestion or bloody or purulent discharge. Physical examination upon admission revealed a soft neck, negative meningeal irritation, and no sinus area tenderness. There were elevated CRP, ESR, anti-protease 3 (PR3) antibody (Table 1). Antinuclear extract antibody, complement, immunoglobulin anti-cyclic citrullinated peptide antibody, immunoglobulin G4 (IGg4), and ferritin exhibited no obvious abnormalities. Infection indices: no abnormalities in the giant cell, herpes virus, procalcitonin, T-SPOT.tuberculosis (T-SPOT.TB) test or tumor markers. The upper lobe of the right lung was flaky and dense on a chest CT (computed tomography), with multiple granular high-density radial artifacts. Dynamic enhancement of cranial MR + diffusion-weighted imaging inner ear MR plain scan: Soft tissue appeared around the nasopharynx, and bone destruction occurred next to soft tissue. Bulbotoiditis in the middle ear of both ears (Fig. 1). Contrast-enhanced CT of the nasopharynx showed the presence of soft tissue surrounding the nasopharynx (Fig. 2).

**Table 1:** The change in clinical and laboratory manifestations.

	October 6, 2022	October 14, 2022	January 12, 2023	July 06, 2023	September 14, 2023
Headache (VAS)	10	5	10	0	0
WBC (4–10 × 10^9^/L)	14.5	7.8	7.3	5.0	7.7
Hb (110–150 g/L)	116	115	100	130	134
Plt (100–300 × 10^9^/L)	397	311	345	256	272
RBC (3.5–5.5 × 10^9^/L)	4.3	4.2	3.4	4.6	4.6
ESR (0–20 mm/h)	80	40	84	3	17
CRP (0–6 mg/L)	66.2	2.8	53	0.5	5.1
Anti-PR3 antibody (0–20 RU/mL)	99.48	34.88	-	9.33	-
Globulin (20–35 g/L)	25.4	29.6	22.6	20.7	21.7
CD19 + B Cells (7–22%)	-	25.75	-	0	0
NK Cells (10–19.8%)	-	25.43	-	22.68	25.11

CRP = C-reactive protein, ESR = erythrocyte sedimentation, Hb = hemoglobin, NK Cells = natural killer cells, Plt = platelet, RBC = red blood cell, VAS = visual analog scale, WBC = white blood cell.

### 2.2. Therapeutic intervntions

**Figure 3.**
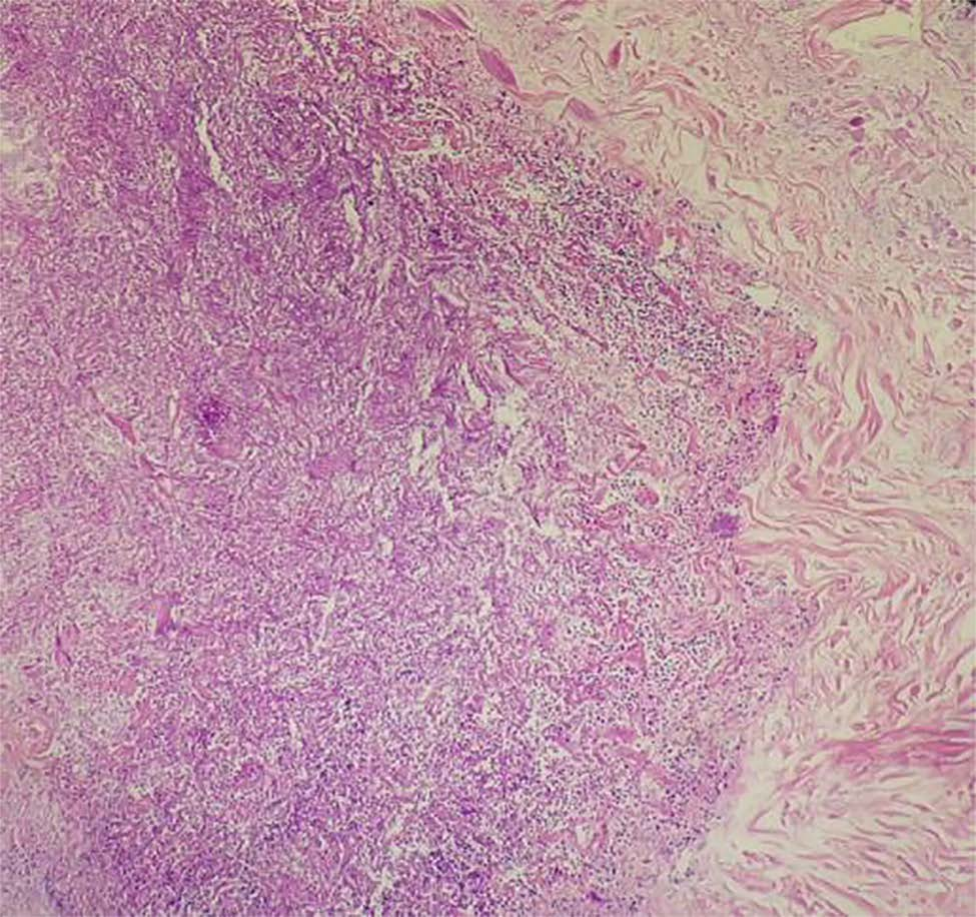
Most of the nasopharyngeal necrotic tissue sent for examination was necrotic tissue. On the surface, bacterial flora was detected, indicating the presence of chronic inflammation of mucosal tissue in nasopharyngeal tissue(H&E × 200).

We believed the patient had central nervous system damage caused by GPA. Therefore, the patient received methylprednisolone pulse therapy (500 mg for 4 days) combined with cyclophosphamide (CTX 500 mg, intravenous infusion l~2 weeks) to alleviate symptoms. The patient headache symptoms improved 3 days after reducing methylprednisolone to 80 mg/bid in combination with 20 g/qd of gamma globulin. Nasopharyngeal pathological biopsy revealed necrotic tissue and bacterial flora on the surface. It indicated the presence of chronic inflammation of mucosal tissue in nasopharyngeal tissue (Fig. 3). Retesting CRP were normal, but the level of anti-PR3 antibody and ESR were slightly elevated (Table 1). Methylprednisolone was gradually tapered from 60 mg bid for 3 days, to 40 mg/bid for 3 days, to 40 mg in the morning and 20 mg in the afternoon for 3 days, and to 40 mg/qd for 15 days. Meanwhile, CTX and compound trimoxazole were applied sequentially to prevent disease recurrence. The cumulative amount of CTX was 2.6 g. A repeat chest CT revealed nodules in the right lung upper lobe and the appearance of cavitary shadows. Considering the patient relapse, the methylprednisolone was adjusted again to 40 mg/bid, in which he responded well to the dose increase. The chest CT was reviewed 2 weeks later, and the cavitary shadow in the upper lobe of the right lung had diminished. The patient headache symptoms improved with methylprednisolone reduction and sequential administration of cyclophosphamide, and he was discharged from the hospital.

### 2.3. Follow-up and outcomes

After the gradual titration of glucocorticoids, the patient reexperienced headache attacks and the cumulative amount of CTX was 5.0 g on January 12, 2023 (Table 1). After receiving 375 mg/m^2^ of rituximab per week for 4 weeks, the patient received 500 mg after 6 months and did not experience headache and hearing loss. The current daily prednisone acetate dosage is 10 mg qd and anti-PR3 antibody, CRP, ESR, VAS returned to normal.

## 3. Discussion

Patients with GPA are believed to have: granulomatous lesions originating in the brain parenchyma or meninges, granulomatous lesions adjacent to the sinuses or other sinus cavities invading the meninges and cranial nerves, and cerebral vasculitis-related.^[5]^ The clinical features of this case are as follows: the patient was an elderly man with a subacute onset and chronic course; multiple systems were involved in the progression of the disease; inflammatory indicators were elevated; and glucocorticoid therapy was effective. Blood and cerebrospinal fluid etiological analysis ruled out most infectious diseases, and the patient exhibited no clinical manifestations of respiratory, urological, or neurological infections. Glucocorticoid therapy was significantly improved to exclude infectious diseases. Cranial imaging, tumor markers, and a nasopharyngeal pathological biopsy ruled out the presence of malignant tumors, including craniocerebral tumors, nasopharyngeal carcinoma, and nasopharyngeal lymphoma. The patient had ear, nose, and lung involvement, and laboratory results revealed high titers of anti-PR3 antibodies. The headache was caused by central nervous system damage caused by GPA, which was a precise diagnosis.

Headache is the most common clinical manifestation of GPA in hypertrophic dururitis. Localized or diffuse dural thickening manifesting as meningogranulomatous lesions characterize this condition.^[6,7]^ Pathologic immunoassay showed fibrosis with infiltration of numerous inflammatory cells, including neutrophils, monocytes, and macrophages, and expression of numerous inflammatory factors and chemokines in serum and cerebrospinal fluid, including T-helper 1 (Th1) cells and interleukin 6 (IL-6).^[8,9]^ The most sensitive imaging examination, skull-enhanced MRI, shows thickening and obvious enhancement of the dura.^[10]^ In this case, the MRI-enhanced T1 sequence did not show focal or diffuse dural thickening, and there was insufficient diagnostic evidence for hypertrophic thick meningitis. Likewise, the literature does not support the presence of negative imaging and meningitis associated with hypertrophic predural manifestations.

Fragoulis GE et al^[11]^ discovered that the pathogenesis was associated with subsequent central nervous system vasculitis in GPA patients with ear, nose, and throat as an early manifestation.^[12,13]^ The middle ear, facial nerve, and dura mater are anatomically adjacent, and inflammatory granulomas in the tympanic cavity can cause facial nerve paralysis by destroying the facial nerve canal. On the other hand, because the middle ear vein communicates with the dural vein, small vessel vasculitis and inflammatory mediators in the middle ear can directly spread to the dura mater.^[14]^ The preceding finding suggests that granulomatous lesions adjacent to sinuses or other sinus cavities can invade the meninges and cranial nerves. However, in this case, the patient early manifestation was a headache, and not an ear, nose, and throat involvement; the above pathogenesis cannot account for the early onset of severe headache.

The dura mater comprises 2 layers of dense, fibrous connective tissue; the outer layer is close to the skull, and the inner layer is the meningeal layer. The nerve terminal structures of the dura mater respond to stretching, with each stretch causing intense pain, particularly in the sensory nerve fibers accompanying the arteries, which are more sensitive to pain. Pain location and nature vary based on the extent of meningeal involvement. Sinus disease and bone erosion frequently accompany local orbital GPA.^[15]^ Imaging studies have found that bone erosion occurs not only in the orbital wall adjacent to granulomas but also in the nasal septum and nasal cartilage, suggesting that local inflammation of inflammatory granulomas leads to bone destruction and directly causes pain.^[16]^ In this case, the nasopharyngeal mass resembles a typical involvement of nasopharyngeal carcinoma. The lateral wall of the nasopharynx and the pharyngeal crypt are the most common sites of nasopharyngeal carcinoma, and headache and cranial nerve damage, which can manifest as persistent migraines with fixed sites, are the most common neurological manifestations. It may be a headache caused by tumor destruction of the base of the skull or spread of the said base involving cranial nerves. By analogy with GPA, local vascular inflammation and local bone destruction at the base of the skull may lead to severe headache.

## 4. Conclusions

Central nervous system involvement is a rare manifestation of active-phase localized GPA. Awareness-raising, early diagnosis and prompt treatment are the keys to halting disease progression and reducing permanent nerve damage. This article reported one case of GPA presenting with headache. The patient early headache could not be explained by hypertrophic dura meningitis or localized granulomatous lesions that invaded the central nervous system. Patients with severe headaches likely have vascular inflammation and local bone destruction at the base of the skull.

## Author contributions

**Funding acquisition:** Xuebin Wang.

**Investigation:** Zhao Li, Qiushuang Zhang, Fang Shi.

**Resources:** Fang Shi.

**Supervision:** Qiushuang Zhang, Xuebin Wang.

**Writing – original draft:** Zhao Li.

**Writing – review & editing:** Zhao Li, Xuebin Wang.
